# Factors associated with the use of health services by elderly men in Brazil: a cross-sectional study

**DOI:** 10.1186/s12889-019-7232-0

**Published:** 2019-07-02

**Authors:** Alana Maiara Brito Bibiano, Vanessa de Lima Silva, Rafael da Silveira Moreira

**Affiliations:** 1 Department of Collective Health, Aggeu Magalhães-Fiocruz Research Center, Av. Prof. Moraes Rego, S/N, Federal University of Pernambuco Campus, Cidade Universitária, Recife, Pernambuco 50670-420 Brazil; 2 Department of Speech and Hearing Therapy, Federal University of Pernambuco, Av. Prof. Moraes Rego, 1235, Cidade Universitária, Recife, Pernambuco 50670-420 Brazil

**Keywords:** Health services, Health of the elderly, Men’s health

## Abstract

**Background:**

Studies have shown that older men use health services less frequently than women do. However, there has been little scientific research on this subject, making it necessary to investigate the influence of various factors on the profile of health service use by this population. This study aims to analyze both the profile of health service utilization by elderly Brazilian men and associated factors.

**Method:**

A population-based cross-sectional study with a secondary database from the National Health Survey (2013) were used. A total of 10,536 male individuals aged 60 years or over were included. The dependent variable was composed of the research questions related to the Use of Health Services, grouped and categorized through the Latent Class Analysis. The independent variables were the factors of predisposition, capacity and health need, organized according to a theoretical model. The association measures were performed by the Rao-Scott test and those of effect by simple and multiple models of multinomial logistic regression. The level of significance was 5%.

**Results:**

The use of health services was marked by the predominance of sporadic medical appointments in the year previous to the study. It was observed that elderly men from the North, Northeast and Midwest regions, those who were illiterate, those without private health insurance, those who had been diagnosed as having a disease, those with functional difficulties and those with a perception of their health status as very poor had been reluctant to use health services of medium and high complexity in the previous 2 weeks, or accept hospitalization. In the previous year, they had consulted their doctor only sporadically.

**Conclusion:**

It was noted that unfavorable social issues affected the profile of health service utilization and that the health care of the elderly Brazilian man is centered on the disease and on curative and rehabilitative attention. In this context, intra- and inter-sectorial policies and actions should encourage early contact of the elderly male population with the health services, especially Primary Care services.

## Background

The concept of use includes all direct or indirect contact with the health services, from medical consultations and hospitalizations to preventive and diagnostic tests [1]. The classic theoretical model of Service Utilization is that of Andersen and Newman [2], which addresses the use of health services as dependent on individual determinants grouped with the factors of predisposition, capacity and necessity, in which predisposing factors influence the capacity and needs represent the most proximal determinant of the use of health services [3, 4].

Necessity factors are related to people’s subjective perceptions and health status. The capacity factors refer to the ability of an individual to seek and receive health services, directly linked to economic conditions and the provision of services: income, health insurance, family support, availability, proximity and quantity of services offered. Predisposing factors refer to individual characteristics that may increase the chance of health service use, such as sociodemographic and family variables: age, sex, schooling and race [3, 4].

In Brazil, some studies have shown that men use health services less frequently than women, in general owing to gender variations in needs. Women use services more for gynecological and obstetric issues and perceive health risks more easily than men because they have more access to information [5–7].

In contrast, men display more chronic health conditions and die more frequently than women from the main causes of death [8–10]. In the field of production of knowledge on men’s health, studies have evolved to minimize discrepancies in the health of the male population relative to the female, to reflect on health inequalities, and to point out the importance of a broader view of the differences between men and women [11].

It is worth noting, however, that this production of knowledge is centered on the male population between 20 and 59 years of age [11–14]; men over 60 are still underexplored in scientific research, making it necessary to investigate the various epidemiological, socioeconomic and cultural factors that influence health and the profile of health service use by this population.

A systematic review [15] of the factors associated with the use of health services by elderly men was conducted in 2017 and the authors included eight articles that identified different profiles of service use, such as hospitalization, screening for prostate cancer, use of mental health services, among others, and that health need factors such as clinical diagnoses and functional disabilities were the most associated with the use.

Each article included in the review [15] searched for a different type of health service and associated factor, not considering the complexity of determining the use of health services, since this is a phenomenon not directly observed or measured through traditional approaches to statistical analysis.

Thus, there is still a gap in the knowledge about the analysis of this theme, and this article, in turn, had the objective of analyzing the factors associated with the profile of health services utilization by elderly men in Brazil through the Latent Class Analysis, an innovative statistical methodology in the area of health that transcends traditional statistical analysis.

## Methods

### Study design and data

This cross-sectional, population-based study from Brazil uses the secondary database of the Brazilian National Institute of Geography and Statistics (*Instituto Brasileiro de Geografia e Estatística* or IBGE, in Portuguese) of the National Health Survey (*Pesquisa Nacional de Saúde* or PNS), which addresses the health of the population of Brazil in three published volumes [16–18].

### Sampling and participants

We included elderly individuals (60 years old or more) from the males selected by the sampling process of the PNS-IBGE. Only elderly men with information missing from the database were excluded.

The PNS is domicile-based and a three-stage sampling plan, with stratification of Primary Sampling Units (PSUs), was used. The census sectors or set of sectors made up the PSUs, the households formed the second-stage units and the residents aged 18 years and over defined the third-stage units to respond to the survey interview. The selection of each of the three stages was carried out by Simple Random Sampling. Details of the design and sample selection process can be found in the PNS reports [16–19].

According to the latest demographic census (2010), 10.8% of Brazil’s population is elderly and 4.8% are elderly men. In the PNS, 60,202 households were visited in Brazil (2013) with interviews conducted with individuals aged 18 years or over. Of the total of 205,546 individuals who answered the PNS, 23,815 (11.5%) were elderly, and, of these, 10,541 (5.1%) were male. Considering the complex sampling design, we used the weights and strata needed to ensure the correct accuracy of the estimates.

### Variables and measures

The dependent variable in this study corresponds to the health service profile used by elderly men and comprised ten questions from the PNS regarding the use of health services (Table 1). These ten questions were grouped and categorized through Latent Class Analysis (LCA), a statistical method that identified distinct mutually exclusive groups (latent classes) based on the response patterns of the ten categorical variables [20]. Following analysis, these were presented as a single variable of health service use that represents a variety of phenomena in order to explain the outcomes.

**Table 1:** Description of dependent and independent variables related to National Health Survey questions, 2019

Variable - issue of PNS	Response category
Dependent variable	
Do you usually seek the same place, same doctor or even health service when you need health care?	Yes
	No
When did you last see a doctor?	In the last 12 months
	From 1 year to less than 2 years ago
	From 2 years to less than 3 years ago
	3 years or more ago
	Never went to the doctor
When did you last see a dentist?	In the last 12 months
	From 1 year to less than 2 years ago
	From 2 years to less than 3 years ago
	3 years or more ago
	Never went to the dentist
In the last 2 weeks, have you looked for a place, service or health professional for health-related care?	Yes
	No
What was the main reason you sought health care in the past 2 weeks?	Accident or injury - disease - dental problem
	Rehabilitation or therapy - continuation of treatment - complementary diagnostic examination
	Vaccination - other preventive care - request for health certificate
	Other
Where did you look for the first health care for this reason in the last 2 weeks?	Pharmacy - other service
	Public Service - primary care
	Public Service - secondary and tertiary care
	Private Service
In the last 12 months, were you hospitalized for 24 h or more?	Yes
	No
What was the main health care you received when you were hospitalized (for the last time) in the last 12 months?	Clinical management
	Psychiatric treatment
	Surgery
	Investigations
	Other
The health facility where you were last hospitalized in the last 12 months was:	Public
	Private
	Do not know
In the last 12 months, did you have emergency care at home?	Yes
	No
Independent variables - Necessity factors	
Has a doctor diagnosed any chronic, physical or mental illness or long-term illness (more than 6 months in duration)?	Yes
	No
In general, what degree of difficulty do you have in eating alone with a dish placed in front of you, including holding a fork, cutting food and drinking in a glass?	Cannot
	With great difficulty
	With some difficulty
	No difficulty
In general, what degree of difficulty do you have in taking a shower alone, including getting in and out of the shower or bath?	Cannot
	With great difficulty
	With some difficulty
	No difficulty
In general, what degree of difficulty do you have in going to the bathroom alone including sitting and lifting the toilet?	Cannot
	With great difficulty
	With some difficulty
	No difficulty
In general, what degree of difficulty do you have in dressing yourself, including putting on socks and shoes, and closing and opening buttons and zippers?	Cannot
	With great difficulty
	With some difficulty
	No difficulty
In general, what degree of difficulty do you have in walking alone from one room to another in the house, on the same floor, such as from the bedroom to the living room and kitchen?	Cannot
	With great difficulty
	With some difficulty
	No difficulty
In general, how difficult is it to lie down or get out of bed alone?	Cannot
	With great difficulty
	With some difficulty
	No difficulty
In general, what degree of difficulty do you have in sitting or standing up by yourself?	Cannot
	With great difficulty
	With some difficulty
	No difficulty
In general, what degree of difficulty do you have in shopping alone, for example for food, clothing or medicine?	Cannot
	With great difficulty
	With some difficulty
	No difficulty
In general, how difficult is it to manage finances on your own (taking care of your own money)?	Cannot
	With great difficulty
	With some difficulty
	No difficulty
In general, how difficult is it to take medicine alone?	Cannot
	With great difficulty
	With some difficulty
	No difficulty
	Does not use medicines
In general, how difficult is it to go to the doctor alone?	Cannot
	With great difficulty
	With some difficulty
	No difficulty
In general, what degree of difficulty do you have in getting around using a bus, subway, taxi, car, etc.?	Cannot
	With great difficulty
	With some difficulty
	No difficulty
In the last 12 months, have you had a fall that prompted you to go to the health department?	Yes
	No
In general, how is your state of health?	Very good
	Good
	Fair
	Bad
	Very bad
Independent Variables - Capacity Factors	
Do you have any health plans, medical or dental, private, company or public agency?	Yes
	No
How long have you had this health insurance?	Up to 6 months
	Over 6 months up to 1 year
	Over 1 year up to 2 years
	Over 2 years
	I have no health insurance
What do you think of this health plan:	Very Good
	Good
	Fair
	Bad
	Very bad
	Never used the health plan
	I have no health insurance
Do you participate in organized social activities (clubs, community or religious groups, day centers for the elderly etc.)?	Yes
	No
Independent variables - Predisposition factors	
In which region of the country are you resident?	North
	Northeast
	Southeast
	South
	Midwest
Condition at home	Person responsible for the home
	Person not responsible for the home
Age	Above median
	Below median
Color or race	White
	Black
	Yellow – Indigenous
	Brown
Marital Status	Married
	Separated or Judicially Disqualified
	Divorced
	Widowed
	Single
Can you read and write?	Yes
	No

Source: Prepared by the author

Legend: *PNS* National Health Survey

This statistical approach worked on heterogeneous data in which individuals were classified in the group by similar characteristics. It is assumed that individuals come from the same population and that the trajectory can be extrapolated to an entire population, just as the covariates that affect the trajectory will influence each individual in the same way [21].

The latent classes or trajectories aim to estimate the size and number of latent classes, the probability of each individual’s response – given that it belongs to a certain class –, and to attribute latent class association to individuals in the population [22].

The independent variables were the PNS questions regarding the factors of predisposition, capacity and health needs of the elderly men, organized and adapted according to the classic theoretical model of Health Services Utilization proposed by Andersen and Newman [2].

The variables related to predisposition, capacity and health need factors are described in Table 1. With regard to the factors of necessity, we present some of the validated instruments [23–25] to evaluate difficulty in performing Basic and Instrumental Activities of Daily Living (BADLs and IADLs), but not all questions used in these instruments were used in the PNS, such as urinary and fecal incontinence and difficulty in using the telephone. The set of questions that evaluated BADLs and IADLs could not therefore be grouped according to the already validated instruments. Consequently, the 12 variables related to the degree of difficulty in performing BADLs and IADLs in the PNS were also studied using the LCA statistical method to analyze the variables, group the data according to similar responses and form a single variable for level of difficulty in carrying out BADLs and IADLs.

### Statistical analysis

To evaluate the latent class model and to identify the number of classes that best define the study object, some statistical criteria were considered. The first is entropy, the probability that the individual is perfectly classified in a given latent class, whose measurements can vary between 0 and 1. The closer to 1 the value is, the better the model will be, indicating a good classification of the individual in the class [26].

Other criteria were considered, such as the Akaike Information Criterion (AIC), Bayesian Information Criterion (BIC) and adjusted BIC, used to evaluate model adjustments. In the analysis, the lower the values of AIC, BIC and adjusted BIC, the more suitable the model will be [27]. To evaluate the evolution of the test model, the Vuong, Lo, Mendell, Rubin likelihood test and Lo, Mendell, and Rubin likelihood test criteria were used considering values of *p* < 0.05 as statistically significant.

In this paper, five models with two, three, four, five and six latent classes were tested to identify the number of classes that best represent the object of study according to the statistical criteria mentioned above. The weights and strata of the database for the LCA were considered.

In the descriptive statistical analysis, the quantitative variable corresponding to age was presented by means of central tendency and dispersion, and the Confidence Interval (CI) of 95% was calculated. Qualitative variables were presented in the form of a frequency table and respective CIs of 95%.

**Fig. 1:**
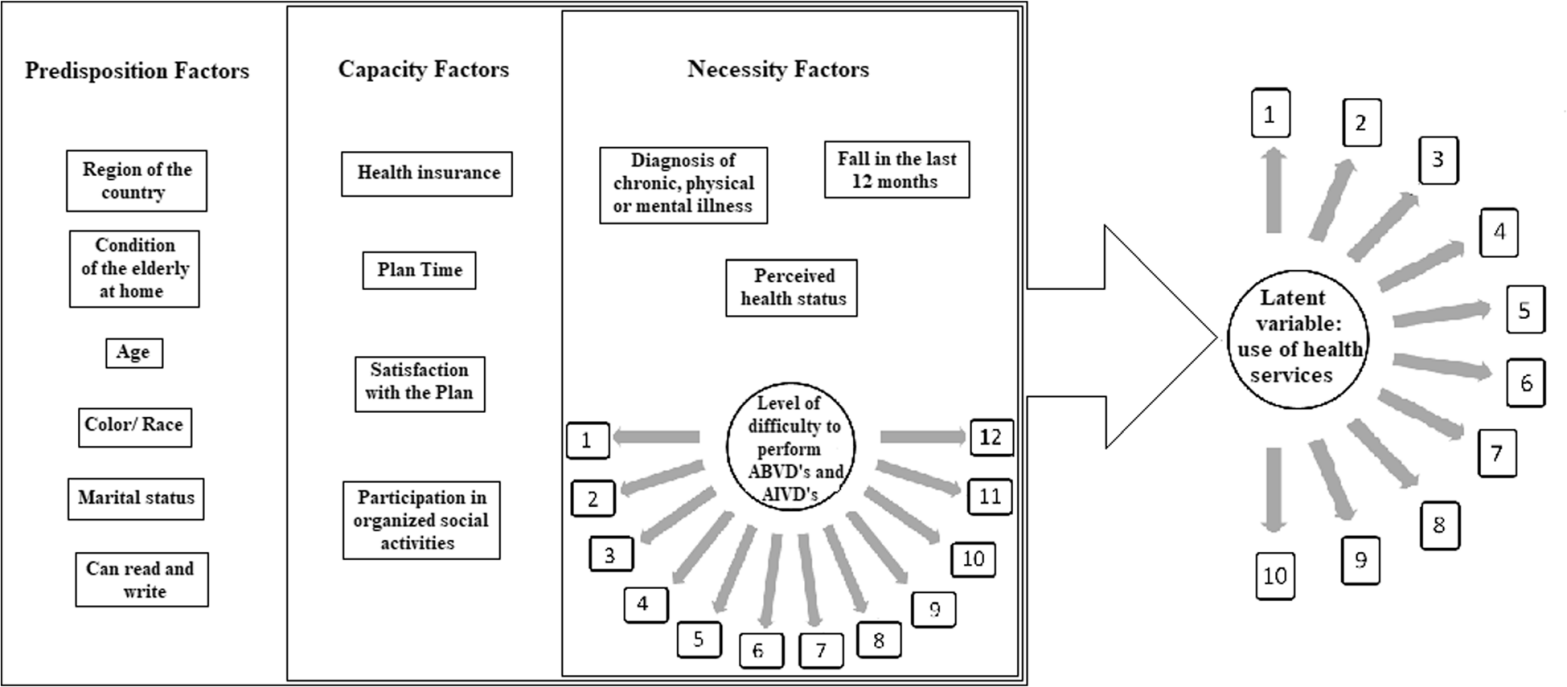
Hierarchical model of analysis of the Use of Health Services by Brazilian elderly men, 2019. Legend: BADLs: Basic Activities of Daily Living; IADLs: Instrumental Activities of Daily Living. Source: Prepared by the author

In the statistical analysis, the presence of association between the independent variables and the dependent variable (categorized by means of Latent Class Analysis) was investigated using the Rao-Scott test used in complex samples [28], which is equivalent to the chi-square test. The level of significance was 5%, and standardized residual values of > 1.96 were considered. The measures of effect of the factors studied on the dependent variable were expressed by the Odds Ratio (OR) and calculated by simple and multiple models of multinomial logistic regression, following the theoretical model of Utilization of Health Services proposed by Andersen and Newman [2] from the assumption of the hierarchical approach of Victora, et al. [29] (Fig. 1).

Initially, a simple analysis was performed on the blocks of factors of predisposition, capacity and health need. Within each block, the variables with *p* < 0.25 [30] were tested in multiple models. At the end, the variables with *p* < 0.05 remained in the final model of each block and were considered adjustment factors for the subsequent blocks.

The statistical programs used were IBM SPSS Statistics, version 20, 2011 for data analysis and Mplus 7.31 to institute latent classes in LCA.

### Ethical aspects

The PNS was approved by the National Commission for Ethics in Research for Human Subjects of the National Health Council, with registration number 328159 on 26th June 2013. The survey participants’ Free Informed Consent forms were signed on the interviewers’ handheld computers. The research plan of this article was exempt from submission to the Research Ethics Committee since it had been supported by secondary data in the public domain.

## Results

### Use of health services

Five models were tested in the LCA to identify the number of classes that, statistically, would best represent the use of health services. The model that established four latent classes was the one that showed the best entropy, adjustment criteria and evolution of the test model, in addition to greater parsimony in the regression model (Table 2).

**Table 2:** Results of suitability and adjustment of the tested latent class models, 2019

Statistical Criteria	Number of classes				
	2	3	4	5	6
AIC	103,492.82	92,281.90	89,290.02	88,772.99	88,488.22
BIC	103,892.26	92,884.69	90,096.16	89,782.48	89,701.07
Adjusted BIC	103,717.48	92,620.93	89,743.42	89,340.76	89,170.36
Entropy	1.000	1.000	1.000	0.936	0.941
Vuong-Lo-Mendell-Rubin – LRT	*p* = 0.0000	*p* = 0.0000	*p* = 0.0000	*p* = 0.0000	*p* = 0.3337
Lo-Mendell-Rubin – LRT	*p* = 0.0000	*p* = 0.0000	*p* = 0.0000	*p* = 0.0000	*p* = 0.3358

Source: Prepared by the author

Legend: *AIC* Akaike Information Criterion, *BIC* Bayesian Information Criterion, *LRT* Likelihood Test

We observed that the models with two, three and four classes showed the same entropy and *p*-values. However, since the values of AIC, BIC and adjusted BIC were the smallest in the model with four classes, this one was selected to represent the use of health services. The four classes identified were titled according to the response patterns of the variables, namely: Use of medium and high complexity health services in the last 2 weeks (4.5, 95% CI 3.9–5.3); Use of health services only for hospitalization in the last year (5.9%; 95% CI: 5.3–6.7); Use of health services for consultation in basic care (17.3, 95% CI 16.2–18.5); Use of health services for sporadic medical visits (one medical consultation in the last year) (72.3, 95% CI 70.9–73.6).

In the first class were included the elderly men who generally seek the same place/doctor for health care; the majority of men who have sought a doctor/dentist in the past 12 months and who have sought health care in the last 2 weeks, mainly due to accident or injury, illness or dental problem, and that the service sought was not primary care. Also included in this class were men who, in addition to using the aforementioned services, were hospitalized in the last 12 months to perform clinical treatment in a public health establishment and those who had emergency at home.

In the second class were included men who usually seek the same place/doctor for health care; the majority of men who sought a doctor/dentist in the last 12 months, and the health service used was only hospitalization, for clinical treatment, in a public health establishment. In this class no man has sought the health service in the last 2 weeks.

In the third class were included the elderly men who usually seek the same place/doctor for health care; the majority of men who have sought a doctor/dentist in the last 12 months and health service in the last 2 weeks, mainly due to accident or injury, illness or dental problem, in primary care. In this class, no man was hospitalized or had an emergency at home in the last 12 months.

In the last class were included men who occasionally seek the same place/doctor for health care; men who have sought a doctor/dentist in the last 12 months, but have not used health services in the last 2 weeks, nor have they been hospitalized or had an emergency at home in the last 12 months.

### Basic and instrumental activities of daily living

In the analysis of the Basic and Instrumental Activities of Daily Living (BADLs and IADLs), five models were also tested in the LCA. The model that established two latent classes was the one that showed the best entropy and evolution of the test model. The first class was entitled “Without difficulty in performing BADLs and IADLs” (86.5%, CI 95% 85.4–87.5) and the second class was entitled “With difficulty in performing BADLs and IADLs” (13.5%, CI 95% 12.5–14.6).

### Descriptive analysis

Of the 10,541 elderly Brazilian men who answered the PNS, 10,536 were included in this study because they had complete information in the IBGE database. The majority were from the Southeast Region (47.6%), followed by the Northeast Region (25.2%), and were responsible for the residence in which they lived (74.0%). The median age was 68 years (1st quartile 63 years and 3rd quartile 74 years) and 52.7% of the subjects were below the median age. The most frequent color/race was white (53.5%), followed by brown (37.0%); while 69.5% were married and 78.8% were literate.

With regard to private health insurance, 71.3% did not have a health plan and, of the 28.7% who did, 26.0% had had the plan for more than 2 years, while 15.0% considered the health plan to be good. The majority of these elderly men did not participate in organized social activities (80.3%), had no diagnosis of chronic, physical or mental illness (64.4%), had no difficulty in performing BADLs and IADLs (86.5%) and had not fallen in the past year (94.5%). The perceptions of good health (41.7%) and regular health (40.1%) were the most frequent.

Table 3 shows the results of the descriptive and analytical analysis of the use of health services by elderly men according to the three blocks of variables analyzed.

**Table 3:** Descriptive and analytical analysis of the Use of Health Services by Elderly Men, Brazil 2019

Variable	Use of services for consultation in basic care % (95% CI)	Use of health services in the last 2 weeks % (95% CI)	Use of services only for hospitalization in the past year % (95% CI)	Use of services for sporadic medical consultation % (95% CI)	*P* value
Block 1 - Predisposition factors					
Region of the country					0,00^*^
North	11,5 (9,5-13,8)	4,5 (3,2-6,2)	5,6 (4,2-7,3)	78,5 (75,8–80,9)^a^	
Northeast	12,7 (11,2-14,3)	3,2 (2,5-4,1)	7,1 (5,8-8,6)	77,0 (75,0-79,0)^a^	
Southeast	20,1 (18,1-22,3)^a^	4,4 (3,3-5,7)	4,1 (3,2-5,3)	71,4 (69,0-73,7)	
South	20,2 (17,8-22,8)^a^	7,0 (5,1-9,5)^a^	8,0 (6,2-10,3)^a^	64,8 (61,0–68,4)	
Midwest	13,0 (11,0-15,4)	5,0 (3,7-6,8)	9,9 (7,8-12,4)^a^	72,1 (69,0-75,0)	
Condition of the elderly at home					0,14
Head of household	17,3 (16,1-18,7)	4,2 (3,4-5,1)	5,6 (4,9-6,5)	72,9 (71,3–74,4)	
Not responsible for home	17,1 (15,0-19,5)	5,5 (4,2-7,2)	6,8 (5,5-8,3)	70,6 (67,7-73,2)	
Age					0,00^*^
Below the median	16,7 (15,2-18,4)	3,6 (2,9-4,5)	5,2 (4,4-6,1)	74,5 (72,5–76,4)^a^	
Above the median	17,9 (16,3-19,7)	5,6 (4,6-6,6)^a^	6,7 (5,8-7,9)^a^	69,8 (67,8–71,7)	
Color / Race					0,00^*^
White	19,0 (17,3-20,8)^a^	4,9 (4,0-6,0)	5,7 (4,8-6,6)	70,4 (68,4-72,4)	
Black	17,8 (14,1-22,1)	5,1 (3,0-8,7)	3,4 (2,2-5,3)	73,7 (68,8–78,0)	
Yellow / Indigenous	19,3 (12,3-28,9)	2,5 (0,7-8,5)	4,8 (1,8-12,3)	73,4 (63,2–81,5)	
Brown	14,6 (13,0-16,4)	3,9 (3,1-4,9)	6,9 (5,7-8,2)^a^	74,6 (72,5–76,6)^a^	
Marital status					0,35
Married	17,2 (15,9-18,7)	4,7 (3,9-5,6)	5,3 (4,6-6,1)	72,8 (71,2–74,4)	
Separated / judicially disqualified	19,9 (13,7-28,1)	5,3 (2,7-10,3)	4,8 (2,5-9,2)	69,9 (60,7-77,8)	
Divorced	17,5 (12,5-24,0)	5,4 (2,9-9,8)	8,5 (5,4-13,3)	68,5 (61,7-74,7)	
Widower	16,6 (13,5-20,3)	3,8 (2,5-5,7)	8,1 (6,1-10,5)	71,5 (67,5–75,2)	
Single	17,5 (14,5-20,9)	3,7 (2,5-5,5)	7,0 (5,1-9,5)	71,8 (68,0-75,4)	
Can read and write					0,00^*^
Yes	18,3 (16,9-19,7)^a^	4,7 (4,0-5,5)	5,3 (4,6-6,0)	71,8 (70,2–73,4)	
No	13,7 (11,7-16,0)	3,9 (2,8-5,5)	8,4 (6,7-10,4)^a^	74,0 (71,1–76,7)	
Block 2 - Capacity Factors					
Health insurance					0,00^*^
Yes	19,6 (17,3-22,0)^a^	5,6 (4,3-7,2)	7,2 (5,8-8,9)^a^	67,6 (64,6-70,5)	
No	16,4 (15,1-17,8)	4,1 (3,4-4,9)	5,4 (4,7-6,2)	74,2 (72,6-75,7)^a^	
Plan Time					0,00^*^
Does not have health insurance	16,4 (15,1-17,8)	4,1 (3,4-4,9)	5,4 (4,7-6,2)	74,2 (72,6-75,7)^a^	
Up to 6 months	16,7 (8,4-30,6)	4,7 (2,8-7,7)	8,8 (2,9-23,9)	69,8 (54,0–82,0)	
More than 6 months to 1 year	13,4 (4,8-32,4)	1,1 (0,2-5,1)	5,5 (1,2-22,4)	80,0 (58,1–92,1)	
More than 1 year up to 2 years	31,4 (19,7-46,1)^a^	2,8 (0,9-8,5)	2,5 (1,2-5,2)	63,4 (49,2–75,5)	
More than 2 years	19,2 (16,8-21,8)	5,9 (4,5-7,7)^a^	7,5 (6,0-9,3)^a^	67,4 (64,2–70,5)	
Satisfaction with the Plan					0,00^*^
Does not have health insurance	16,4 (15,1-17,8)	4,1 (3,4-4,9)	5,4 (4,7-6,2)	74,2 (72,6-75,7)^a^	
Very good	13,0 (9,6-17,4)	8,1 (4,8-13,5)	9,8 (6,3-15,1)	69,1 (62,2–75,2)	
Good	20,4 (17,2-24,0)	5,2 (3,8-7,2)	6,8 (5,2-9,0)	67,6 (63,5–71,4)	
Regular	25,0 (19,9-30,8)^a^	5,8 (3,9-8,6)	6,1 (3,8-9,6)	63,1 (56,7–69,1)	
Bad	9,4 (4,0-20,2)	2,6 (0,5-11,5)	14,3 (6,3-29,0)	73,8 (58,9–84,7)	
Very Bad	7,0 (3,1-15,2)	0,5 (0,1-3,9)	2,6 (0,7-8,9)	89,9 (80,1–95,1)^a^	
Never used the plan	20,0 (7,9–42,2)	1,1 (0,2-6,8)	1,5 (0,3-6,1)	77,5 (56,2–90,2)	
Participation in organized social activities					0,59
Yes	18,1 (15,6-21,0)	4,4 (3,1-6,2)	6,7 (5,2-8,7)	70,8 (67,4-73,9)	
No	17,1 (15,8-18,4)	4,6 (3,9-5,3)	5,7 (5,0-6,5)	72,6 (71,1–74,1)	
Block 3 - Factors of Need					
Diagnosis of chronic, physical or mental illness					0,00^*^
Yes	23,2 (21,2-25,4)^a^	7,9 (6,6-9,5)^a^	7,7 (6,5-9,1)^a^	61,1 (58,6–63,6)	
No	14,0 (12,8-15,4)	2,6 (2,1-3,3)	4,9 (4,2-5,8)	78,4 (76,9-79,9)^a^	
Level of difficulty in performing BADLs and IADLs					0,00^*^
No difficulty	17,1 (15,9-18,4)	3,2 (2,6-3,9)	5,2 (4,5-5,9)	74,5 (73,0-76,0)^a^	
With difficulty	18,5 (15,5-21,8)	12,9 (10,3-16,2)^a^	10,8 (8,8-13,3)^a^	57,8 (53,6–61,9)	
Fall in the last 12 months					0,00^*^
Yes	23,0 (17,6-29,4)	10,9 (7,8-14,9)^a^	13,1 (9,5-17,6)^a^	53,1 (46,5–59,6)	
No	17,0 (15,8-18,2)	4,1 (3,5-4,9)	5,5 (4,8-6,3)	73,4 (71,9-74,8)^a^	
Perceived health status					0,02^*^
Very Good	17,4(13,5-22,2)	4,4 (2,4-8,1)	9,0 (6,1-13,0)	69,2 (63,4-74,4)	
Good	18,0 (16,2-19,9)	4,0 (3,1-5,1)	5,7 (4,8-6,8)	72,4 (70,2–74,4)	
Regular	16,9 (15,3-18,7)	4,7 (3,8-5,8)	5,7 (4,7-6,9)	72,6 (70,5–74,7)	
Bad	16,7 (13,4-20,7)	4,4 (2,9-6,6)	6,4 (4,6-8,8)	72,5 (68,1–76,5)	
Very Bad	13,3 (8,7-19,9)	11,6 (6,9-19,0)^a^	2,8 (1,1-7,1)	72,2 (64,4-78,9)	

Source: Prepared by the author

Legend: *95% CI* 95% Confidence Interval, *BADLs* Basic Activities of Daily Living, *IADLs* Instrumental Activities of Daily Living

^*^p < 0.05 (Rao and Scott test)

^a^Standard Residues> 1.96

### Multiple logistic regression

The results of the final multiple multinomial logistic regression model after adjustments of the three blocks of variables are shown in Table 4. The use of consultation services in primary care was considered as the reference category of the dependent variable.

**Table 4:** Adjusted odds ratio values and confidence intervals obtained by multinomial logistic regression analysis, 2019

Variable	Use of health services in the last 2 weeks OR (95% CI)	Use of services only for hospitalization in the past year OR (95% CI)	Use of services for sporadic medical consultation OR (95% CI)	*P* value
Block 1 - Predisposition factors^a^				
Region of the country				0,00^*^
North	1,09 (0,59-1,99)	1,03 (0,62-1,71)	2,03 (1,51-2,74)^†^	
Northeast	0,71 (0,41-1,23)	1,15 (0,76-1,73)	1,78 (1,39-2,28)^†^	
Southeast	0,61 (0,38-0,97)^†^	0,52 (0,35-0,78)^†^	1,10 (0,89-1,38)	
South	1,00	1,00	1,00	
Midwest	1,08 (0,64-1,82)	1,80 (1,15-2,80)^†^	1,68 (1,29-2,18)^†^	
Age				0,00^*^
Below the median	1,00	1,00	1,00	
Above the median	1,48 (1,10-2,00)^†^	1,13 (0,85-1,51)	0,84 (0,71-1,01)	
Can read and write				0,03^*^
Yes	1,00	1,00	1,00	
No	1,02 (0,65-1,59)	1,64 (1,16-2,32)^†^	1,19 (0,94-1,50)	
Block 2 - Capacity Factors^b^				
Satisfaction with the Plan				0,00^*^
Does not have health insurance	0,99 (0,58-1,67)	0,99 (0,57-1,71)	1,61 (1,16-2,26)^†^	
Very Good	2,61 (1,18–5,74)^†^	3,25 (1,50-7,04)^†^	2,19 (1,36–3,55)^†^	
Good	1,07 (0,58-1,97)	1,38 (0,75-2,55)	1,33 (0,91-1,94)	
Regular	1,00	1,00	1,00	
Bad	1,12 (0,17-7,25)	5,48 (1,39–21,55)^†^	3,13 (1,19–8,26)^†^	
Very Bad	0,34 (0,04-2,99)	1,54 (0,33–7,06)	4,95 (1,89–12,95)^†^	
Never used the plan	0,20 (0,02-1,77)	0,24 (0,04-1,41)	1,52 (0,50-4,63)	
Block 3 - Factors of Need^c^				
Diagnosis of chronic, physical or mental illness				0,00^*^
Yes	1,48 (1,06-2,05)^†^	0,88 (0,66-1,18)	0,50 (0,43-0,59)^†^	
No	1,00	1,00	1,00	
Level of difficulty in performing BADLs and IADLs				0,00^*^
No difficulty	1,00	1,00	1,00	
With difficulty	3,36 (2,20–5,13)^†^	1,74 (1,21-2,50)^†^	0,83 (0,64-1,06)	
Fall in the last 12 months				0,00^*^
Yes	1,39 (0,81-2,38)	1,47 (0,91-2,36)	0,55 (0,38-0,79)^†^	
No	1,00	1,00	1,00	
Perceived health status				0,01^*^
Very Good	1,24 (0,59-2,59)	1,83 (1,07-3,12)^†^	1,01 (0,72-1,44)	
Good	1,00	1,00	1,00	
Regular	1,26 (0,89-1,77)	0,97 (0,72-1,31)	1,00 (0,84-1,20)	
Bad	0,95 (0,54-1,69)	1,03 (0,65-1,65)	1,06 (0,78-1,44)	
Very Bad	3,39 (1,57-7,32)^†^	0,49 (0,17-1,44)	1,24 (0,75-2,03)	

Reference Variable: Use of the Health Service for consultation in basic care

Source: Prepared by the author

Legend: *OR* Odds Ratio, *CI 95%* 95% Confidence Interval, *BADLs* Basic Activities of Daily Living, *IADLs* Instrumental Activities of Daily Living

^*^p < 0.05; ^†^variable category with *p* < 0.05

^a^Adjusted by the variables of Block 1 - Predisposition Factors

^b^Adjusted by the variables of Blocks 1 - Predisposition Factors and Block 2 - Capacity Factors

^c^Adjusted by the variables of Blocks 1 - Predisposition Factors, Block 2 - Capacity Factors and Block 3 - Necessity Factors

In the multiple logistic regression of Block 1, accepting the reference category of the “South” region of Brazil, the elderly men from the North had approximately 2 times, the Northeast 1.8 times and the Midwest 1.7 times the probability of having used the services for sporadic medical consultation in relation to consultation for basic care; those in the Southeast were 39% less likely to have used the services in the previous 2 weeks and 48% less likely to have been hospitalized; while those in the Midwest were 1.8 times more likely to have been hospitalized than to have made primary care consultations.

With respect to age, men over 68 years (reference category “below the median”) were 1.5 times more likely to have used the services in the previous 2 weeks compared to the reference category (primary care referral). In addition, those who did not know how to read or write (the “read and write” category) were 1.6 times more likely to have been hospitalized in the previous year than those of reference category.

In the analysis of Block 2, after adjustment of the variables in Block 1, only the variable of satisfaction with the health plan (“regular” reference category) had an effect on the use of health services. The elderly men who considered their health plan “very good” were 2.6, 3.2 and 2.2 times more likely to have used the services in the previous 2 weeks, to have undergone hospitalization in the previous year and to have made sporadic medical consultations, respectively, than to have made consultations in basic care. Those who considered the health plan “bad” were 5.5 times more likely to have been hospitalized and 3.1 times more likely to have made a sporadic medical visit; while those who considered the plan “very bad” were 4.9 times more likely to have seen the doctor sporadically than to have consulted one for basic care.

In the last block, after adjustments of the variables with the statistical significance of the previous blocks, those who had been diagnosed with a chronic, physical or mental disease were 1.5 times more likely to have used the services in the previous 2 weeks than those who had not; and had 50% less chance of using the service sporadically than the reference category. Those who showed difficulty in performing BADLs and IADLs (reference category “without difficulty”) were 3.4 times more likely to have used the services in the previous 2 weeks and 1.7 times more likely to have been hospitalized in the previous year in relation to consultation in basic care.

Elderly men who reported a fall in the past year (reference category “had no fall in the past 12 months”) showed a 45% probability of less than occasional medical consultation in relation to the primary care visit. Older men who reported a “very good” health status (“good” category) were thus 1.8 times more likely to be hospitalized, and those who reported “very bad” were 3.4 times more likely to have used the services in the previous 2 weeks than to have made a primary care visit.

## Discussion

According to the World Aging and Health Report, an aging population demands a comprehensive public health response. However, discussion of the subject has been insufficient and evidence limited, calling for urgent action to explore the issue [31]. With regard to the aging of the male population, the situation becomes even more worrying, since research is even scarcer.

The profile of health service utilization by elderly Brazilian men was analyzed using LCA. This established four distinct classes of use based on the response patterns to the categorical variables of the PNS and enabled a study of the phenomenon that encompasses its various nuances through a novel yet secure statistical methodology that has rarely been used in epidemiological studies. It is noteworthy that there have been no previous studies to investigate the use of health services by elderly men using ACL methodology.

The studies found on this theme defined the dependent variable by using a single question, such as: “When did you last go to any health service?” [32]; “What type of health service do you use most often in terms of management/financing?” [13]; “Number of consultations with health professionals in the last 12 months” [33]; “Have you consulted professionals (for example, doctors, psychotherapists) for depressive symptoms?” [34], among others, which did not address the phenomenon in its totality and complexity, as investigated in this study through LCA.

With regard to the latent classes generated, we identified that most of the subjects had used the health service for sporadic medical consultations in the previous year (72.3%), that is, they are not frequent users. Only 17.3% had used the services for consultation in Primary Care, and a minority had been hospitalized in the last year or used medium and high complexity services in the previous 2 weeks.

According to the National Basic Attention Policy [35], Primary Care should be the user’s preferred contact in the Brazilian health system, the main gateway and communication center of the Health Care Network. This was not the case among elderly Brazilian men, and it is emphasized that the procurement of health care by this population usually occurs in extreme situations or at specialized levels of the health system [36]. In Canada, according to a review of the situation of men’s health in the country, it was found that refusal to seek medical care reached 80%, which could lead to an increase in hospital morbidity rates [37].

In the hierarchical analysis of Block 1 - Predisposing Factors, the elderly men of the North, Northeast and Midwest were more likely to use services for sporadic medical visits, while those in the Midwest had a higher probability of being hospitalized than to have primary care consultations. The geographical distribution and the local availability of the service network can produce barriers to the use of health services [33], especially those of Primary Care consultation.

In contrast, the elderly men in the Southeast, the richest region of the country, had a protective factor in using the services in the previous 2 weeks and being hospitalized. This can be explained by the fact that more egalitarian regions have greater social capital, which is related to the greater use of health services, possibly owing to the local population’s superior information network concerning the health system [33].

With regard to age, men over 68 years were 1.5 times more likely to have used the services in the previous 2 weeks compared to the reference category (primary care referral). This data reveals that being older in Brazil is a predictor of having used medium- and high-complexity services in the previous 2 weeks. It should be pointed out that the population of 80 years and older, even though it is the smallest of the age groups, is the one that has been increasing most rapidly over the years and requires a differential health approach to meet the needs of this specific population [38].

Similar findings were reported in the United States study that examined the use of three types of health services by older men: admission to skilled nursing care, admission to a hospice and hospitalization. It concluded that the older (85 years or older) the individual, the greater the chance of him using the health services [39].

Elderly literacy was also assessed, and it was observed that men who did not know how to read or write were 1.6 times more likely to have been hospitalized in the last year than in primary care. A study carried out in the Republic of Korea found that older men who had only primary or non-school education had a 30% lower probability of consulting a mental health professional [34].

According to inequality studies that used the database of Health, Welfare and Aging in Latin America [40, 41], elderly people with poorer education have a worse health status owing to worse habits, greater exclusion and less information and socioeconomic conditions for early access to the health network, leading to the use of services in more severe health conditions, such as those requiring hospitalization.

In the analysis of Block 2 - Capacity Factors, elderly men in Brazil who did not have a health plan were 1.6 times more likely to use the medical consultation services sporadically in relation to primary care consultation. In the Republic of Ireland [42], a study analyzed the determinants of prostate cancer screening in elderly men and identified having health insurance as a positive factor in taking the Prostate Specific Antigen (PSA) test.

The level of satisfaction with the health plan is an indirect measure or proxy to have a health plan, because only those who are satisfied or not with the plan, is who owns it. In this study, this variable had an effect on the use of health services. Those who were dissatisfied with their health plan were more likely to be hospitalized or referred to the doctor sporadically, while those who considered the health plan “very good” were more likely to have used the services in the previous 2 weeks, undergone hospitalization in the last year and had sporadic medical consultation rather than primary care consultation.

It was observed that despite extreme degrees of (dis) satisfaction (“very good”, “bad” or “very bad”) with the health plan having an effect on use, the worst indicators of hospitalization and sporadic medical consultation were still among those who considered the plan more negatively.

Regarding the last block - Necessity Factors, the elderly men who had a diagnosis of chronic, physical or mental illness, in relation to those who did not, were 1.5 times more likely to have used the services in the previous 2 weeks and had 50% less chance of seeing their doctor sporadically. This shows that seeking a health professional coincides with a moment of discomfort generated by some ongoing symptom or disease [43] and reveals that clinical diagnosis is conditioned by the frequent use of health services.

Similar findings were found in studies conducted in the Republic of Korea [34] in The United States [39] and Australia [44] that evaluated several types of health services used by elderly men, and identified that a medical history of several diseases and comorbidities greatly determined the use of health services, especially in consulting a mental health professional, admission to a specialized nursing center, admission to a hospice and hospitalization.

The prevalence of functional limitation varies among countries and according to the criterion adopted for its definition [45, 46]. A widely used definition is the reporting of difficulties in carrying out BADLs and IADLs. In this study, older men who had difficulty performing BADLs and IADLs were 3.4 times more likely to have used the services in the previous 2 weeks and 1.7 times more likely to have been hospitalized in the previous year than to have made a primary care visit.

These data corroborate the Spanish study that prospectively examined the relationship between functional status and the use of a wide variety of health services among the older adult population and identified that limitation in performing IADLs had an adverse risk effect on the use of the various health services studied: influenza vaccination, home services, primary care medical consultation, emergency services and hospitalization [47].

Regarding the occurrence of falls among elderly men, those who had suffered some type of fall in the previous year showed a 45% lower chance of having a sporadic medical consultation than a primary care consultation. In this study, the fall had no risk effect on the use of health services in the previous 2 weeks nor on hospitalization in the previous year, but it has already been stated in the literature that falls among the elderly have an association with hospitalization [48–50].

Finally, perceptions of health, understood as interpretations that individuals make about their own health, have been used in large-scale surveys and have been understood as an important indicator for, among other things, how individuals perceive their well-being. Elderly men in Brazil who reported a “very good” perception were 1.8 times more likely to be hospitalized; in the United States study, individuals who were 1.75 times more likely to be hospitalized than those who had reported the perception of “bad” health [51].

Those who considered their health “very bad” were 3.4 times more likely to have used the services in the previous 2 weeks, similar to the study in the Republic of Korea [34] that analyzed 1827 elderly men and observed that poor subjective health status was associated with consulting health professionals, demonstrating that the worse the perception of health, the greater the use of services.

The data of the three blocks of factors of need, capacity and predisposition for health care did not show many variations when comparing the same variables studied by the National Health Survey by Household Sample (*Pesquisa Nacional por Amostra de Domicílios* or PNAD) in the years of 1998, 2003 and 2008, research carried out before the PNS to study health in Brazil [52]. This demonstrates the reliability of the data that the PNS has produced when it sets out to study exclusively the health of the Brazilian population. It is a good model for the use of data in scientific research.

As a methodological limitation, this study demonstrated some restrictions that are common in surveys that use secondary databases, namely: the variables studied in the PNS database were already established, which prevented new variables from being included; the primary research objectives were distinct from current research and prevented new information from being acquired; and, long questionnaires, such as those applied in the PNS, can generate memory bias, in which the participant forgets or loses the desire to report past events.

Limitations on the design of the cross-sectional study were also present. All the variables that composed the latent classes were asked at the same time and demanded that the respondent remember the time of use of the health services. However, there was no way to know the reason for use (causality), but to identify patterns of use based on the types and times of use of health services by elderly men.

However, despite the intrinsic limitations of the methodological design, this article is an essential contribution to the study of aging in the male population, with the possibility of introducing a new perspective on the subject and serving as a planning tool for public policy institutions and actions concerning elderly Brazilian men.

## Conclusion

The use of health services by elderly Brazilian men was marked by the prevalence of sporadic medical consultations in the past year, a departure from the desire to use primary care services that should be the main gateway to health services for the entire population, including the elderly male public.

In the hierarchical analysis, the blocks of factors of predisposition, capacity and health need had an association with and an effect on the use of health services. It was observed that was associated with using the services in the last 2 weeks: older people, satisfied with the health plan, who had a diagnosis of chronic disease, difficulty to perform BADLs and IADLs and who had very bad health perception. It was associated with hospitalization in the last year: elderly people living in the Midwest, who did not know how to read or write, very good or bad satisfaction with the health plan, who had difficulty performing BADLs and IADLs and very good perception of health status. Finally, it was associated with sporadic use of the service: elderly people from the north, northeast and center-west, who had no health plan, and those who had, were satisfied with the plan, and that the presence of clinical diagnosis of chronic or fall factors were protective factors for sporadic use.

This indicates that the profile of health service utilization by these individuals is influenced by unfavorable social conditions and that the health care of elderly Brazilian men is centered on disease and curative and rehabilitative care rather than on health promotion and prevention of diseases. In this context, intra- and inter-sectoral policies and actions should encourage the contact of the elderly male population with the health services at an earlier stage, especially in Primary Care.

We therefore recommend that research on human aging should also be focused on the male population, in order to increase the health knowledge of this population and to establish goals and actions to improve its health indicators.

## Data Availability

This study uses the secondary database of the Brazilian National Institute of Geography and Statistics (Instituto Brasileiro de Geografia e Estatística or IBGE, in Portuguese) of the National Health Survey (Pesquisa Nacional de Saúde or PNS). The datasets generated and/or analyzed during the current study are public and are available in the IBGE repository, [http://www.ibge.gov.br].

## Abbreviations

AIC: Akaike Information Criterion
BADLs: Basic Activities of Daily Life
BIC: Bayesian Information Criterion
CI: Confidence Interval
IADLs: Instrumental Activities of Daily Life
IBGE: Brazilian National Institute of Geography and Statistics or *Instituto Brasileiro de Geografia e Estatística*, in Portuguese
LCA: Latent Class Analysis
OR: Odds Ratio
PNAD: National Health Survey by Household Sample or *Pesquisa Nacional por Amostra de Domicílios*, in Portuguese
PNS: National Health Survey or *Pesquisa Nacional de Saúde*, in Portuguese
PSA: Prostate Specific Antigen
PSU: Primary Sampling Units
